# Improvements to pairwise sequence comparison (PASC): a genome-based web tool for virus classification

**DOI:** 10.1007/s00705-014-2197-x

**Published:** 2014-08-14

**Authors:** Yiming Bao, Vyacheslav Chetvernin, Tatiana Tatusova

**Affiliations:** National Center for Biotechnology Information, National Institutes of Health, Bethesda, MD 20894 USA

## Abstract

The number of viral genome sequences in the public databases is increasing dramatically, and these sequences are playing an important role in virus classification. Pairwise sequence comparison is a sequence-based virus classification method. A program using this method calculates the pairwise identities of virus sequences within a virus family and displays their distribution, and visual analysis helps to determine demarcations at different taxonomic levels such as strain, species, genus and subfamily. Subsequent comparison of new sequences against existing ones allows viruses from which the new sequences were derived to be classified. Although this method cannot be used as the only criterion for virus classification in some cases, it is a quantitative method and has many advantages over conventional virus classification methods. It has been applied to several virus families, and there is an increasing interest in using this method for other virus families/groups. The Pairwise Sequence Comparison (PASC) classification tool was created at the National Center for Biotechnology Information. The tool’s database stores pairwise identities for complete genomes/segments of 56 virus families/groups. Data in the system are updated every day to reflect changes in virus taxonomy and additions of new virus sequences to the public database. The web interface of the tool (http://www.ncbi.nlm.nih.gov/sutils/pasc/) makes it easy to navigate and perform analyses. Multiple new viral genome sequences can be tested simultaneously with this system to suggest the taxonomic position of virus isolates in a specific family. PASC eliminates potential discrepancies in the results caused by different algorithms and/or different data used by researchers.

## Introduction

Viruses are classified based on their properties, such as morphology, serology, host range, genome organization and sequence. With the development of better sequencing technologies, more virus sequences have become available in public databases. In the last ten years (as of June, 2014), the total number of virus sequences in the GenBank database increased from 0.2 million to 1.8 million, and the total number of complete viral genome sequences collected in the National Center for Biotechnology Information (NCBI) Viral Genome Project [1] increased from 1,150 to 3,980. This makes sequence-based virus classification more feasible.

The most commonly used sequence-based virus classification tool is phylogenetic analysis. The classification of about 70 % of the families and floating genera described in the Ninth Report of the International Committee on the Taxonomy of Viruses (ICTV) is supported by phylogenetic trees [2]. However, phylogenetic analysis is usually computationally intensive and requires expertise to interpret the results.

Lauber and Gorbalenya [3] developed a sequence-based virus classification method called DEmARC (*D*iv*E*rsity p*A*rtitioning by hie*R*archical *C*lustering). In this approach, multiple sequence alignment is performed on proteins from all genomes of a virus family, and the pairwise evolutionary distances (PEDs) among all genomes are calculated. The distribution of PEDs is used to quantitatively estimate hierarchy levels of taxonomy in the family. DEmARC is objective and has been applied to the classification in the families *Picornaviridae* [3] and *Filoviridae* [4], but it is not well suited for high-throughput applications.

Recently, a novel method using Natural Vector, based on distributions of nucleotide sequences, was reported to characterize phylogenetic relationships among some viruses [5]. Its application to virus classification has been investigated [6], and high prediction accuracies have been achieved at the genus level and above. Its ability to classify viruses at the species level needs to be improved.

Another sequence-based molecular classification method for viruses is based on pairwise identities of virus sequences within a virus family. A histogram is then generated to represent the number of virus pairs at each percentage of sequence identity. This will usually produce peaks that represent different taxonomic groups such as strains, species, and genera, and the percentages of the lowest points between the peaks can be used as demarcation criteria for different taxa. This method has been applied to a few viral taxonomic groups including the families *Coronaviridae* [7], *Geminiviridae* [8–10]*, Papillomoviridae* [11]*, Picornaviridae* [12], *Potyviridae* [13] and the species *Rotavirus A* [14]. A major drawback of this method is the inconsistency of the results when different protocols are used to calculate the pairwise identities. The exact algorithm and parameters used to establish the demarcation criteria are very difficult for researchers to reproduce when testing their own sequences. The identities obtained from different protocols are therefore not comparable. To overcome this problem, NCBI created a PASC (Pairwise Sequence Comparison) resource [15], where the same protocol is used for both procedures.

We have applied a new algorithm and added several new features to the NCBI PASC tool since it was initially launched, and these will be described in this paper. The new implementations greatly enhance the performance of the tool and improve the results by eliminating artifacts that were associated with the old method.

## Materials and methods

### Source of genome sequences and taxonomy information

For a given virus family/group, complete genome sequences are retrieved from the NCBI viral genomes collection [1], which includes both reference sequences and genome sequences of other members of the same species. These sequences, together with their NCBI taxonomy lineages, are stored in a database. The database is updated every day to add new genome sequences and reflect taxonomy changes.

### Pairwise global genome alignment and identity calculation

For viruses whose genomes are smaller than 32 kb, the alignment is done using the global Needleman-Wunsch alignment algorithm [16] with the affine scoring model. The scores are 1 for matches and -1 for mismatches, gap openings and indels. For viruses with large genomes (>ca. 32 kb), the Hirschberg’s divide-and-conquer algorithm [17] is used to perform the global alignment. This algorithm features the affine gap penalty model and runs in linear space, thereby saving memory. Since genomes can vary in length, terminal gaps are not penalized. However, they are not discounted either when pairwise identities are computed, i.e., a shorter genome perfectly matching a longer one will produce a pair with less than 100 % identity.

### BLAST-based alignment and identity calculation

Two rounds of BLAST [18] are performed on each pair of genome sequences. In the first round, the translated protein sequences of one genome in six frames are searched against the nucleotide sequence of the other genome using tblastn. The amino acid alignments in the tblastn results are converted back to nucleotide alignments. In the second BLAST round, pairwise blastn is carried out on the nucleotide sequences of the genomes. We then select a consistent set of hits from the two sets of BLAST results, giving preference to higher-identity hits and trimming overlaps out of lower-identity hits, to generate a set of hits that do not overlap in any region on any genome. This process will select blastn hits for closely related genomes, but most likely tblastn hits for distant ones. A mixture of blastn and tblastn hits might be used in some cases. The aligned regions used to calculate the identities can be viewed as a matrix plot and detailed alignments in text (see the upper part of Fig. 5 for an example). Since our algorithm does not discriminate between genuine and spurious BLAST hits, nor does it evaluate the likelihood of an open reading frame being expressed *in vivo*, some false BLAST hits might be used. Also, because of the overlap-trimming process, our algorithm creates some artificial tiny hits (as short as 1 nucleotide). But the number of artificial hits is far smaller than that of genuine hits (compare the tiny dots with the lines marked with arrows in the upper plot in Fig. 5) and therefore can be ignored.

Pairwise identities are calculated as the total number of identical bases in local hits divided by the average length of the genome pair.

### Removal of redundant sequences

To increase the speed of the tool, sequences from members of the same species and with identities higher than a predefined value (between 95 and 99.5 % for different viral groups) are represented by one sequence in the dataset. The excluded sequences are referred to here as redundant sequences.

### Identity distribution plot

The identity distribution chart is plotted based on pairwise alignments computed between all members of the selected virus family or group. The pair is represented in green color if both genomes belong to the same species according to their assignment in NCBI’s taxonomy database; in yellow color if the two genomes belong to different species but the same genus; and in peach color if they belong to different genera. Both linear and log scales are available for the y-axis (number of pairs).

### Taxonomy change simulation

We provide a tool to simulate taxonomy changes by changing species and/or genus demarcations to user-provided values. We build a hierarchical tree using complete linkage (furthest neighbor) agglomerative clustering, based on pairwise identity distance (100 % − identity %). We cut this tree at a user-specified level and analyze resultant clusters. For example, to merge species above 90 %, we cut the tree at 90 % identity. If any resultant cluster contains genomes from different species, we merge these species. To separate species below 80 %, we cut the tree at 80 %. If there are species divided by resultant clusters, we divide them accordingly. A list of taxonomy changes necessary to achieve the user-proposed demarcation is provided.

## Results

The PASC resource at NCBI can be accessed through http://www.ncbi.nlm.nih.gov/sutils/pasc. It currently covers 56 virus families/groups, which are listed at http://www.ncbi.nlm.nih.gov/sutils/pasc/viridty.cgi?textpage=main.

**Fig. 1:**
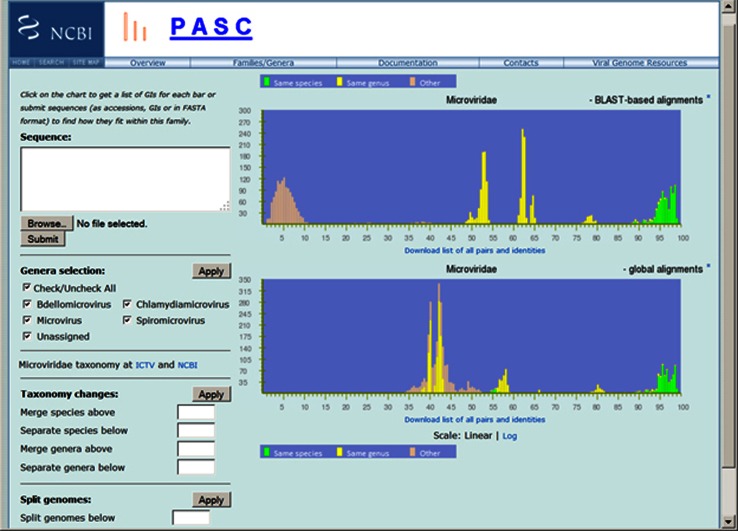
Frequency distribution of pairwise identities from the complete genome sequence comparison of 87 microviruses

Figure 1 shows the PASC result for the family *Microviridae*. The upper plot shows the pairwise identity distribution of 87 genomes calculated from the BLAST-based alignment, while the lower plot is that calculated from the global alignment. We use three colors to label virus pairs with different taxonomic relationships. The green, yellow and peach bars in the plots represent pairs of genomes that are assigned to the same species, to different species but the same genus, and different genera, respectively, in the current NCBI taxonomy database. Clicking a bar reveals a list of genome pairs that form the bar, along with taxonomy positions of the genomes, and their sequence identity.

### Establishing demarcation criteria

The BLAST-based alignment panel in Fig. 1 demonstrates a good PASC result where the bars of different colors group together and the groups are well separated. This indicates that a) the classification based on sequence identities of genomes in the family *Microviridae* agrees mostly with the current ICTV taxonomy; b) the taxonomy assignment of genome sequences within the family in the INSDC databases (DDBJ/ENA/GenBank) are fairly accurate; c) clear species and genus demarcations can be set for the family, which is between 82 % and 85 % for species, and between 28 % and 34 % for genera. It is important to keep in mind that when selecting demarcations, users should focus on the sizes of gaps between the peaks rather than the heights of the peaks. The peak heights are determined by the number of genomes in each species and genus and therefore could be misleading due to sampling bias within particular species/genera. The gap sizes, on the other hand, indicate the possibility that these regions will be filled with sequences from novel viruses. The larger the gaps, the more likely that they are the true threshold to separate species/genera.

### Simulation of taxonomy changes

**Fig. 2:**
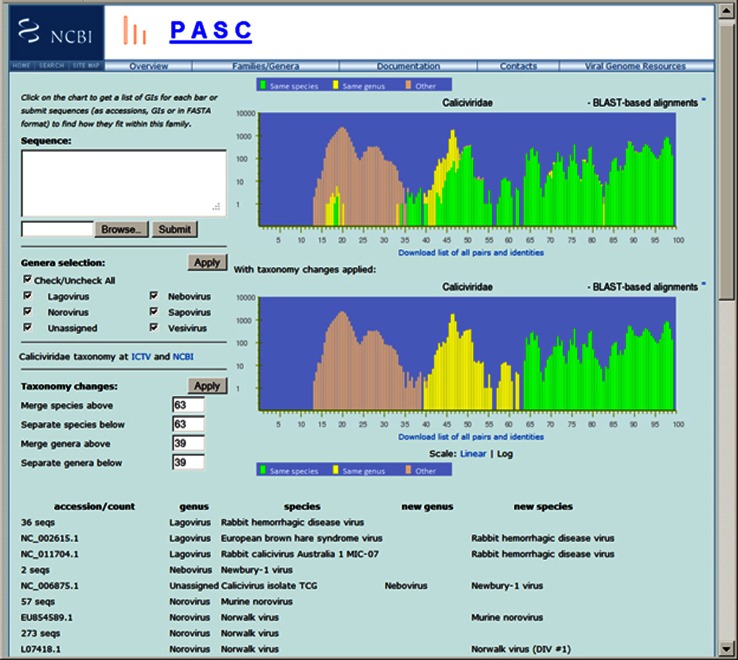
Frequency distribution of pairwise identities from the complete genome sequence comparison of 274 caliciviruses, and the simulation of taxonomy changes using proposed species and genus demarcations

Recently, we added a new feature in PASC that allows users to test ideas for genus/species demarcation and see what taxonomy changes are needed using existing sequences to achieve user-selected demarcations. Figure 2 illustrates how taxonomy changes can be simulated in the family *Caliciviridae*. The upper plot shows the pairwise identity distribution of 274 genomes calculated by BLAST-based alignment. We can see green and yellow bars in the 15-20 % range, mixed with the dominating peach bars; and some yellow bars in the greater than 69 % region, mixed with the dominating green bars. This indicates that some of the viruses might be assigned to an incorrect lineage by GenBank sequence submitters. Also, the current species demarcation criteria are probably too low for the percentage of sequence similarity, because the green bars go all the way down in the region below 40 %, which is not common in other virus families.

To explore different demarcations, users can try different numbers for “Merge species above” and “Separate species below”, and for “Merge genera above” and “Separate genera below” as indicated at the lower left section of “Taxonomy changes:”, namely 63 and 39. As a result, a perfect taxonomy can be achieved when 63 % and 39 % are used as the species and genus demarcation, respectively. The lower plot shows the same genome set after taxonomy changes are applied. In this example, 56 instead of 63 can also be tried for “Merge species above” to see if an alternative species demarcation can be established. When more than one demarcation is acceptable in PASC, other criteria (e.g., host) should be taken into consideration to select the suitable one.

Following the output depicted in Fig. 2, PASC also lists recommended taxonomy changes needed for such a result, some of which are shown at the bottom of Fig. 2. In this example, PASC indicates that both European brown hare syndrome virus and rabbit calicivirus Australia 1 MIC-07 belong to the species *Rabbit hemorrhagic disease virus*, and calicivirus isolate TCG, which is currently unassigned to a genus, belongs to the species *Newbury-1 virus* in the genus *Nebovirus*.

Another taxonomy change simulation tool in PASC splits genomes into subgroups. For example, there is a peak below 12 % in the upper plot in Fig. 1, indicating genome groups with very low similarities. To see how these groups are formed, the number 12 is entered in the box next to “Split genomes below” at the lower left section of Fig. 1. After this is applied, PASC determines that three groups can be formed – the first consists of Cellulophaga phage phi12:2 and Cellulophaga phage phi12a:1, the second consists of the genus *Microvirus*, and the third consists of three genera, *Bdellomicrovirus*, *Chlamydiamicrovirus* and *Spiromicrovirus*, plus two unclassified viruses, Marine gokushovirus and Microviridae phi-CA82. Plots showing the distributions of pairwise similarities of genomes within the three groups are also provided (data not shown). This suggests that three subfamilies can be created from these three groups. Indeed, the ICTV has already assigned the genera *Bdellomicrovirus*, *Chlamydiamicrovirus* and *Spiromicrovirus* to the subfamily *Gokushovirinae*. The genus *Microvirus* should form a second subfamily, and the two other viruses, Cellulophaga phage phi12:2 and Cellulophaga phage phi12a:1, a third.

### How to classify newly sequenced viruses

**Fig. 3:**
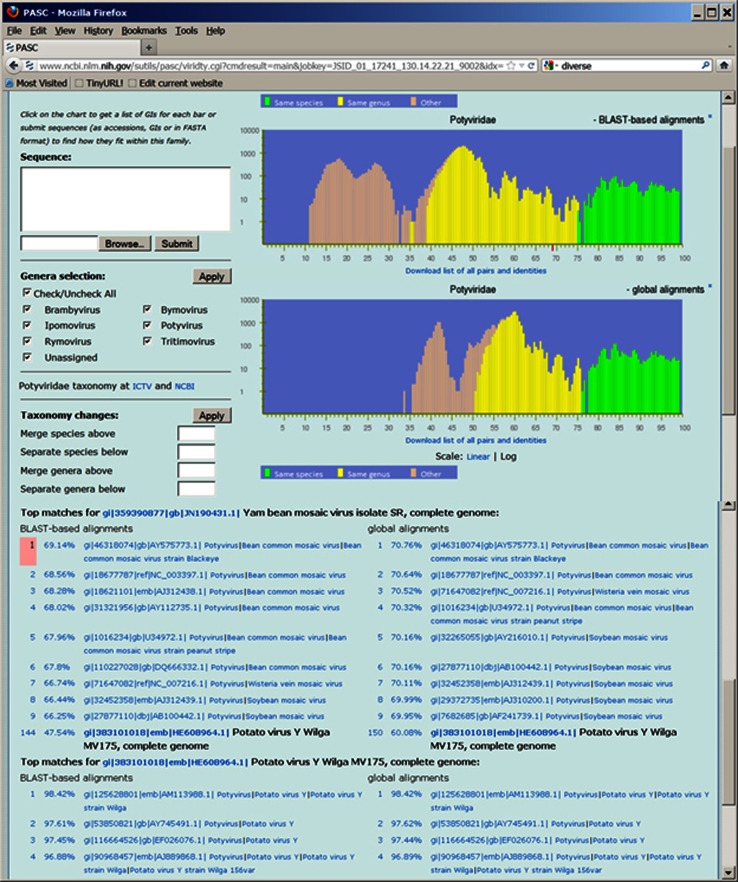
Frequency distribution of pairwise identities from the complete genome sequence comparison of 260 potyviruses and its application in classifying newly sequenced viruses

The primary application of PASC is to classify viruses with newly sequenced genomes. This is demonstrated using the example in Figure 3. The accession numbers of two potyvirus genomes recently released in GenBank, JN190431 and HE608964, were entered into the “Sequence” box of the PASC page for the family *Potyviridae* and compared with existing potyvirus genomes and with each other. For each input genome, PASC produces a list of pairwise identities, from the highest to the lowest, between this input genome and the rest of the input genomes, and 5 to 10 of the closest matches to existing genomes within the family. The identity distribution chart depicts the currently selected genome in a different color. One can click on each genome’s number to make it current, or can click the identity to see details of the alignment.

The closest genome to JN190431 was AY575773 (bean common mosaic virus strain Blackeye), and their identity was 69.14 % by the BLAST-based alignment method. This is indicated by a red bar on the x-axis of the upper plot. Since this red bar is located in the region consisting of yellow bars, this suggests that JN190431 and AY575773 belong to different species but should be in the same genus. The organism name for JN190431, yam bean mosaic virus, is currently a species-level name under the “unclassified potyvirus” node in NCBI’s taxonomy database, which indicates that its classification agrees with the PASC analysis, but this name has not been approved by ICTV.

AM113988 (potato virus Y strain Wilga) was the closest genome to HE608964. It showed 98.42% identity by the BLAST-based alignment method. Since the red bar is in the region consisting of green bars, PASC suggests that HE608964 and AM113988 belong to the same species, and therefore, the organism name for HE608964, potato virus Y, is appropriate.

The identity between the input sequences JN190431 and HE608964 was also reported by PASC, which was 47.54 %. Such a report is very useful in determining the relationships among genomes of members of new species, because they could belong to the same new species (if their identity is higher than 76 %) or different ones (if their identity is lower than 76 %). In this case, the result suggests that JN190431 and HE608964 belong to different species.

When a new genome is known to belong to a member of an existing genus, all other genera can be unchecked from the “Genera selection” section below the sequence input box. New sequences will then only be compared with existing sequences in the selected genus, therefore reducing the time of computation. This is particularly helpful for families with a large number of genome sequences available.

## Discussion

### New features

Different colors are used to represent genome pairs that have different taxonomy relationships (e.g., same species, different genera). This makes it a lot easier to identify demarcation criteria – they are at the borderlines between peaks of different colors. We should point out that the taxonomy relationships are based on their current assignment in the NCBI taxonomy database. Although NCBI uses the official ICTV taxonomy names whenever possible, there are times when GenBank sequence submitters assign their sequences to incorrect virus names, which will cause color mixture in the peaks (e.g., yellow bars in the green-dominant peaks or vise versa). These can be easily spotted, and the taxonomy simulation tool can quickly suggest the correct classification.

The removal of redundant genomes can, in some cases, significantly increase the speed with which new genomes are tested without compromising PASC’s ability to establish demarcation criteria. For example, after removing sequences with identities higher than 95 %, only 75 out of the over 5,000 total genomes are left in PASC for the family *Hepadnaviridae* (as of April, 2014). The cutoff that determines the redundancy is mainly based on the number of genomes and can be adjusted if necessary.

### Limitation of global alignment

Previously, genome similarities were calculated based on pairwise global alignments in PASC. Although this method works well for some virus families/groups, such as papillomaviruses and potyviruses, the results are not very good for others. Our analysis showed several problems with the use of global alignment in PASC.

1. In some viruses with circular genomes, such as the circoviruses, there is an inconsistency in the designation of the first nucleotide of the genome sequences in public databases. For example, the genome of Y09921 is about 765 nucleotides off from the majority of other porcine circovirus 1 sequences, with the latter starting from TAGTATTA in the stem-loop region. This shift in the sequence positions reduces genome similarities when they are calculated based on global alignment. For instance, the genome sequences AF055391 and AY484407 belong to the same species, but their genome similarity is only 66 % by global alignment. The similarity would be much higher, at about 97 %, if the two sequences started from the same position in the genome.
2. In some viruses, particularly those with negative-strand RNA genomes, the opposite strand of the genome is sometimes submitted to the public databases. When genome similarities are calculated based on a global alignment of the opposite strands of two genomes, the result is just wrong and meaningless. For example, the genome sequence of BD091237 was deposited as the genomic strand rather than the usual complementary strand for negative-strand RNA viruses. When this sequence is used directly in global alignment with other genomes, the genome with the highest similarity to it, at 54 %, is GU591771. However, if the opposite strand of BD091237 is used in global alignment with GU591771, their similarity is 98 %.
3. For viruses that are distantly related, the identities obtained by global alignment are usually misleading, because the identity of two random genome sequences of the same size could be as high as 50 %. For example, sequences DQ641708 and EU273817 belong to two viruses in different genera and have only minimum similarities in small regions of the genomes. However, their pairwise identity by global alignment could be as high as 49 %, which obviously does not reflect their real similarity.

### BLAST-based alignment greatly improved PASC

To overcome the problems associated with global alignment, we employed BLAST-based alignment to calculate pairwise identities of genome sequences.

We started with tblastx [18], a translation-based alignment method, on genome pairs to calculate their identities. The procedure was similar to the BLAST-based alignment described in Materials and methods, except that tblastx was used instead of the combination of blastn and tblastn. We later observed that the tblastx alignment did not work well for genomes that do not encode proteins (e.g., viroids). In addition, tblastx does not allow gaps in the alignment and is therefore not optimized for genomes with low similarities.

We then switched to the current method, which uses the combination of blastn and tblastn. It selects the better of the two alignments for the same region in the genomes and therefore effectively applies the most appropriate blast program automatically on protein coding and non-coding regions. This approach captures all possible similar regions among the genomes and has improved the results to some extent in almost all of the virus families/groups we currently have in PASC. In the future, we will discontinue the global alignment method to speed up the tool.

For the family *Microviridae*, whose members have circular genomes, there is a great deal of mixture of color bars in the PASC plot based on global alignment (the lower panel in Fig. 1) because some sequences start at different positions in the genome than others. This is corrected completely when BLAST-based alignment is used (the upper panel in Fig. 1), and bars of the same color all group together.

**Fig. 4:**
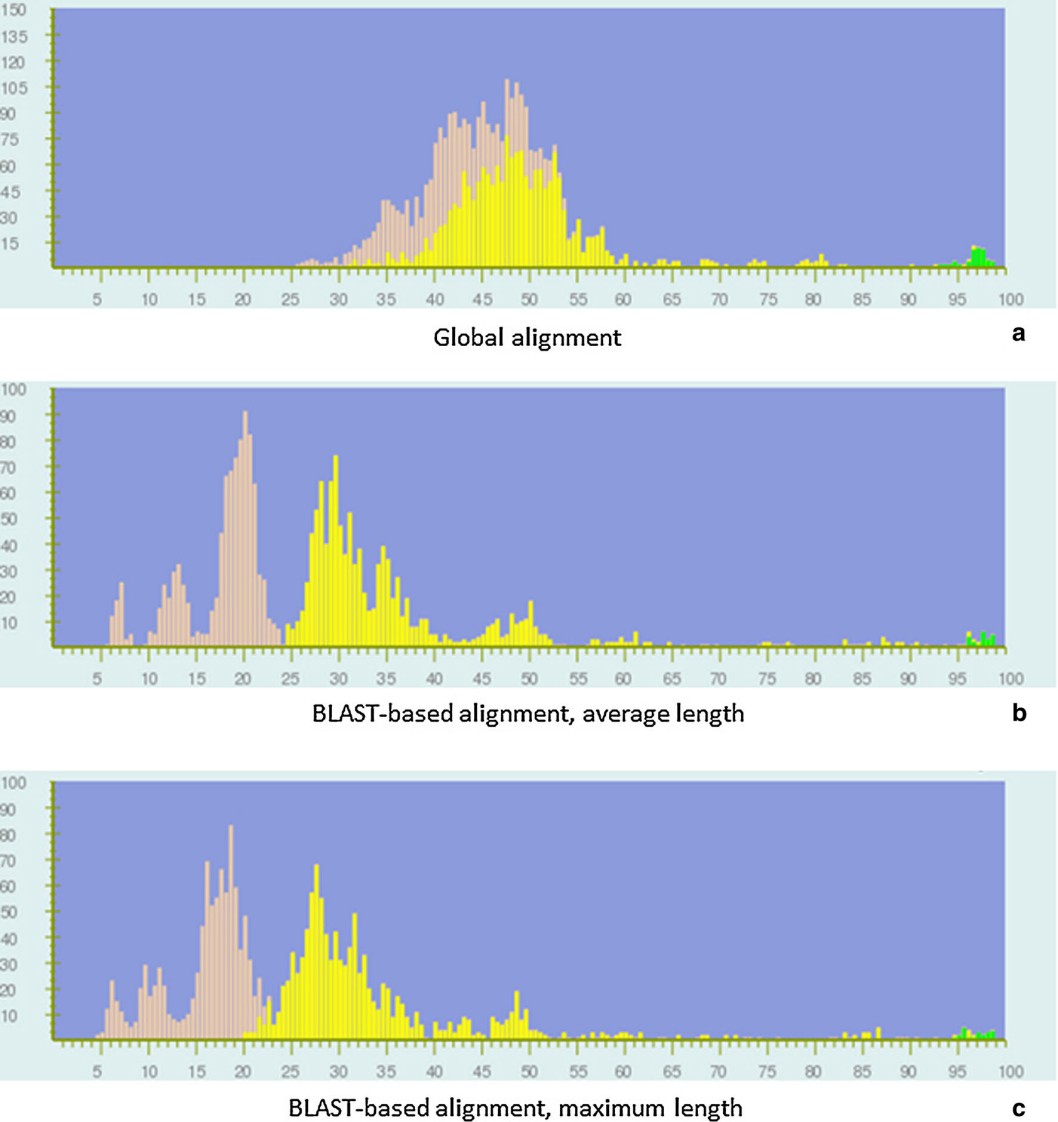
Frequency distribution of pairwise identities from the complete genome sequence comparison of 81 baculoviruses, using global alignment (a) and BLAST-based alignment (b and c). The pairwise genome identities are calculated by the average (b) and maximum (c) length

Previously [15], we discussed that PASC using global alignment does not work well for large genomes with low overall sequence similarities (e.g., the family *Baculoviridae*). This is demonstrated in Fig. 4a, where the yellow and peach bars are mostly mixed. This is greatly improved when BLAST-based alignment is used (Fig. 4b and c). The genome sizes of baculoviruses vary from less than 82 kb to nearly 179 kb. The use of the average genome length (Fig. 4b) instead of the maximum genome length employed previously (Fig. 4c) to calculate pairwise identities also results in better separation of the yellow and peach peaks below 24 % identity.

The BLAST-based alignment method produces better separation for certain taxonomy groups than the global alignment method does. For example, a peak representing groups (i.e., bovine lentivirus group and the primate lentivirus group) in the genus *Lentivirus* is separated from the rest of the yellow peaks, which is otherwise not the case in the global alignment method. For some virus families, demarcation criteria for the sub-species level can be determined by PASC, as demonstrated for marburgviruses [19].

**Fig. 5:**
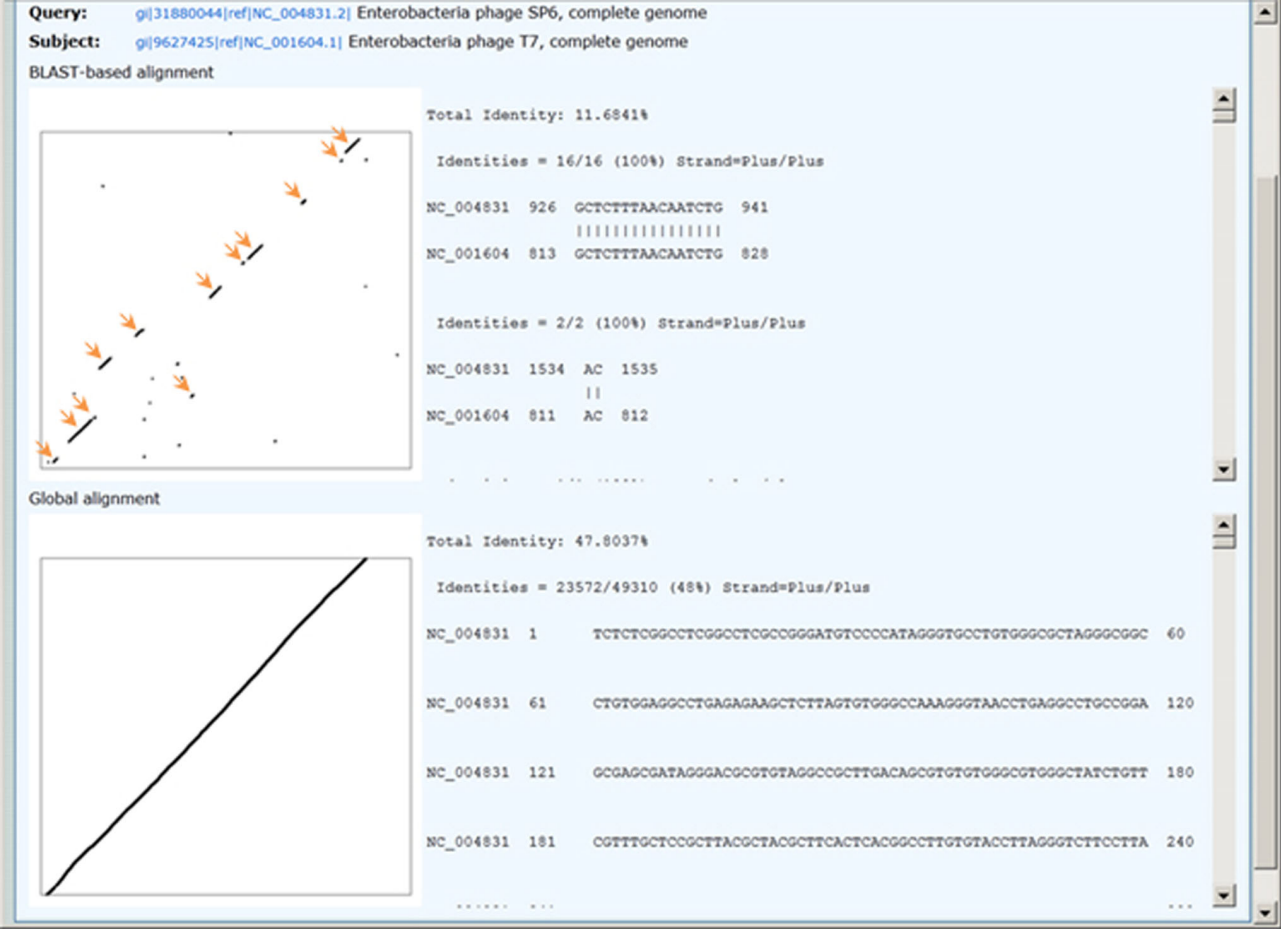
Dot matrix and text views of pairwise alignment between genome sequences of enterobacteria phage SP6 (NC_004831) and enterobacteria phage T7 (NC_001604), using the BLAST-based and global alignment methods. The conserved regions between the two genomes are marked with arrows on the dot matrix from the BLAST-based alignment

The BLAST-based alignment method also makes it possible to apply PASC to some families of phages with large genomes. For the family *Podoviridae*, the overlap of yellow and peach bars below 55 % in the global alignment method no longer exists in the BLAST-based alignment method, and therefore, the genus demarcation criteria can tentatively be set to around 35 %. The genomes of enterobacteria phage SP6 (NC_004831) and enterobacteria phage T7 (NC_001604) are only conserved in the following T7 genes: gp0.3, gp1, gp1.7, gp4, gp5, gp8, gp10, gp11, gp12, gp16 and gp19 [20]. These are the major regions used by the BLAST-based alignment method to calculate the similarity between the two genomes (Fig. 5). This method is essentially the same as the one used by Lavigne et al. [21], where members of the family *Podoviridae* were classified based on similarities of protein sequences. The identity between SP6 and T7 is 12 % using the BLAST-based alignment method here, which is very close to the 15 and 17 % obtained by the two methods described by Lavigne et al. [21]. There are still overlaps between the green, yellow and peach bars in the plot using the BLAST-based alignment. This is mainly because there are currently some unclassified viruses in the NCBI taxonomy database, some of which belong to other official ICTV species. For example, the following unclassified phiKMV-like phages in the NCBI taxonomy database probably all belong to the species *Enterobacteria phage phiKMV*: Pseudomonas phage LKD16, Pseudomonas phage LUZ19, Pseudomonas phage PT2, Pseudomonas phage PT5, Pseudomonas phage phikF77 and Pseudomonas phage vB_Pae-TbilisiM32. These can be identified using the taxonomy change simulation tool described above.

### PASC and other similar methods

It is important to keep in mind that because of the different methods used in calculating the pairwise genome identities, the demarcations obtained using the BLAST-based alignment could be different from those obtained using the global alignment and would be more likely different from those determined by other algorithms using different datasets and/or different genome regions. For example, the species demarcation for the family *Papillomoviridae* is about 65 % and 69 % using BLAST-based and global alignment in PASC (http://www.ncbi.nlm.nih.gov/sutils/pasc/viridty.cgi?cmdresult=main&id=347), respectively. Both are higher than the 60 % currently used by the papillomavirus community, which is based on the L1 gene of these viruses [11]. Therefore, the percentage of identities between existing and new genomes calculated in PASC (e.g., those in Figure 3) is not comparable with other methods and can only be used in this system, and vice versa.

### PASC for segmented viruses

The PASC tool is currently based on complete genomes for non-segmented viruses or on one of the genome segments for segmented viruses (e.g., DNA A of geminiviruses). Working with ICTV’s *Nanoviridae* Study Group, we have added nanoviruses to PASC using their DNA-R, DNA-S, DNA-C, DNA-M and DNA-N segments, and in most cases, PASC assigns a particular nanovirus to the same species regardless of the segment used. However, there are a few instances where species assignment varies depending on the segment used. Further investigations will be needed to find out whether this represents different evolutionary rates in different segments, or e.g., reassortment events among viruses of different species. Many families whose members have segmented genomes are not currently present in PASC, and input from the ICTV Study Groups would be helpful to determine which segment(s) to use.

### PASC and ICTV

The PASC tool is linked to the NCBI’s viral genome collection [1] and taxonomy database, with new viral genomes added and taxonomy status of viruses updated every day. It runs very fast, and results can usually be obtained within minutes. The tool is online, so there is no software to download/install, no parameters to set, and everybody uses the same algorithm and same dataset. This allows for consistent results between different users. We believe that PASC can be a great aid to ICTV Study Groups by providing much more objective criteria for making taxonomic assignments based on sequence comparisons. Indeed, our PASC analysis result for the family *Polyomaviridae* has been adopted by the ICTV Study Group as one of the demarcation criteria for new species in the family. ICTV is the official authority to establish new virus species, but it can take up to several months with the current procedure. When virus sequences are submitted to GenBank, they are immediately required to be placed under a species node (whether an existing species or an unclassified one) in NCBI’s taxonomy database. It is not possible to wait for ICTV to determine what species a sequence belongs to. PASC, on the other hand, can provide a quick guide for the proper species classification, thereby reducing the number of sequences mis-assigned to a species. PASC is routinely used by the NCBI viral genomes group to curate viral genome collections. The taxonomy simulation function of PASC can not only identify problematic entries in the current NCBI taxonomy database but also help the ICTV Study Group to see how demarcation criteria changes affect the taxonomy.

Although the NCBI PASC tool has been used in several studies [22–34], no cutoff values in sequence identity percentages are provided currently in our PASC system that can be used to separate species and genera. We can do this only after the PASC result is accepted by the community to determine the demarcation criteria for a virus family.

There are some families for which peaks overlap in PASC, and therefore, demarcation criteria cannot be easily established (e.g., the family *Betaflexviridae*, data not shown). In such cases, we would like to receive advice from the ICTV Study Groups on alternative ways to perform PASC, e.g., using sequences of one or several genes rather than complete genomes. By working together with the ICTV Study Groups, we believe we can explore the potential of PASC and maximize its application for as many viruses as possible. Any suggestions and comments are always welcome.
